# Sensitivities of Rheological Properties of Magnetoactive Foam for Soft Sensor Technology

**DOI:** 10.3390/s21051660

**Published:** 2021-02-28

**Authors:** Rizuan Norhaniza, Saiful Amri Mazlan, Ubaidillah Ubaidillah, Michal Sedlacik, Siti Aishah Abdul Aziz, Nurhazimah Nazmi, Koji Homma, Shuib Rambat

**Affiliations:** 1 Engineering Materials and Structures (eMast) iKohza, Malaysia-Japan International Institute of Technology (MJIIT), Universiti Teknologi Malaysia, Jalan Sultan Yahya Petra, Kuala Lumpur 54100, Malaysia; r.norhaniza@gmail.com (R.N.); aishah118@gmail.com (S.A.A.A.); nurhazimah@utm.my (N.N.); 2 International Center, Tokyo City University, 1 Chrome-28-1 Tamazutmi, Setagaya, Tokyo 1580087, Japan; khomma@tcu.ac.jp (K.H.); shuibrambat@utm.my (S.R.); 3 Faculty of Engineering, Universitas Sebelas Maret, Jalan Ir. Sutami 36A, Kentingan, Surakarta 57126, Central Java, Indonesia; 4 Centre of Polymer Systems, University Institute, Tomas Bata University in Zlín, Trida T. Bati 5678, 760 01 Zlín, Czech Republic; msedlacik@utb.cz; 5 Disaster Preparedness & Prevention Centre (DPPC), Malaysia-Japan International Institute of Technology (MJIIT), Universiti Teknologi Malaysia, Jalan Sultan Yahya Petra, Kuala Lumpur 54100, Malaysia

**Keywords:** magnetostriction, normal force, porous polymer, magnetic dipolar interaction

## Abstract

Magnetoactive (MA) foam, with its tunable mechanical properties and magnetostriction, has the potential to be used for the development of soft sensor technology. However, researchers have found that its mechanical properties and magnetostriction are morphologically dependent, thereby limiting its capabilities for dexterous manipulation. Thus, in this work, MA foam was developed with additional capabilities for controlling its magnetostriction, normal force, storage modulus, shear stress and torque by manipulating the concentration of carbonyl iron particles (CIPs) and the magnetic field with regard to morphological changes. MA foams were prepared with three weight percentages of CIPs, namely, 35 wt.%, 55 wt.% and 75 wt.%, and three different modes, namely, zero shear, constant shear and various shears. The results showed that the MA foam with 75 wt.% of CIPs enhanced the normal force sensitivity and positive magnetostriction sensitivity by up to 97% and 85%, respectively. Moreover, the sensitivities of the storage modulus, torque and shear stress were 8.97 Pa/mT, 0.021 µN/mT, and 0.0096 Pa/mT, respectively. Meanwhile, the magnetic dipolar interaction between the CIPs was capable of changing the property of MA foam from a positive to a negative magnetostriction under various shear strains with a low loss of energy. Therefore, it is believed that this kind of highly sensitive MA foam can potentially be implemented in future soft sensor systems.

## 1. Introduction

A soft sensor is an advanced technology that is capable of handling small-scale tasks with high accuracy. The technology requires highly compliant, soft, flexible and squishy materials to produce touch devices with high flexibility and sensitivity such as soft grippers, prosthetic hands, human motor systems, biomedical robots, and field robots for data collection [1,2,3]. One of the main components in a soft sensor is a smart material that is capable of measuring physical quantities such as force, displacement, heat, etc., before converting them into an electrical signal, which can then be used in an electrical circuit or microprocessor to generate a readable response for an actuator. Among the few types of smart materials available to date, magnetoactive (MA) foam is one of the potential candidates for use in soft sensor technology due to its flexibility and unique magneto-induced phenomenon that is sensitive to the strength of a magnetic field [4,5,6,7]. MA foam commonly consists of a porous polymer matrix with magnetic particles, usually carbonyl iron particles (CIPs), embedded in it. When the MA foam is exposed to an external magnetic field, the magnetic moments of the CIPs will be induced to realign their poles in the same direction as the external magnetic field pole. During this process, the mechanical and rheological properties of the MA foam, such as its storage and loss moduli, can be controlled and adjusted according to the strength of magnetic field due to changes to the microstructure of the material [8,9,10].

The controllable properties of MA foams have gained a lot of attention for further development through the works of several researchers [8,9,10,11]. Davino et al. [8] prepared MA foam using 5 wt.% to 25 wt.% of iron (Fe) particles with a size of less than 44 µm. The samples were fabricated in anisotropic condition and analysed under compression modes. The MA foam showed a strong dependency on strain as the concentration of iron particles was increased and reached its optimum at 20 wt.%. Under this compression mode, the MA foam showed a linear increase in the stress property, with the magnetic field increasing up to 213 mT. Later, another group of researchers fabricated MA foam with higher concentrations of CIPs, namely, 60 wt.%, 70 wt.% and 80 wt.% with an average particle size of less than 9 µm [9]. Under a shear mode, the sample with 60 wt.% of CIPs showed no dependence on the magnetic field, while the sample with 80 wt.% was dependent on the magnetic field up to a magnetic field strength of 1 Tesla. It is a well-known fact that the particle size influences the density and porosity of any dispersing system, thereby affecting the contact between particles. Since both studies used different sizes and types of magnetic particles, thus, both MA foams exhibited different magnetic properties. However, no details were provided with regard to the magnetic properties of both MA foams.

Meanwhile, concentrations of 5 wt.% to 20 wt.% of two types of magnetic particles, iron (Fe) and barium ferrite (BaFe), with a polyurethane matrix were utilized in the fabrication of MA foams [10]. The results revealed that the MA foam with 20 wt.% of BaFe had a higher Young’s modulus of 1596 kPa compared to the MA foam with 20 wt.% of Fe, which had a Young’s modulus of 1048 kPa, due to the strong magnetism of BaFe compared to iron. Furthermore, the MA foam with 20 wt.% of BaFe revealed a linear trend for the stress with an increase in the magnetic field at a rate of 1 Pa/mT under a compression mode. A similar research under a compression mode was conducted by Volpe et al. [11], where there was an increase in the negative stress with an increase in the magnetic field strength at a rate of 1.5 Pa/mT due to the forces of attraction between the magnetic particles, which caused the sample to shrink. However, was no further discussion to explain the reason behind the negative stress pattern. An analysis of MA foam in a morphological study [9], revealed that the presence of CIPs reduced the size of the pores and increased the nucleation points during the foaming process. However, to date, the relationship between the mechanical properties of MA foam and changes to its microstructure has remained unclear. Besides, most of the researches into MA foam focused and reported on individual physical quantities like stress [8,10,11], storage modulus [9,12,13] or magnetostriction [14,15] as summarized in Table 1.

It is a well-known fact that a combination of different mechanical parameters is crucial for the development of soft sensor technology, particularly in terms of soft sensing capabilities [17,18,19]. Therefore, this study presented a combination of different mechanical performances and sensitivities of MA foam in terms of various physical capabilities such as magnetostriction, normal force, storage modulus, shear stress and torque. The relationship of these properties in association with the morphology of the MA foam was examined to gain further fundamental scientific knowledge and understanding.

## 2. Materials and Methods

For the MA foam, CIPs with an average size of 3 to 5 µm, were supplied by CK Materials Lab. Flexible polyurethane (PU) was used as the foam matrix, where PU polyol and 4-4 MDI diisocyanate were purchased from Smooth On Inc., USA. Three different weight percentages (wt.%) of CIPs, namely, 35, 55 and 75 wt.%, were used in this study. These fractions were calculated from a fixed mass of 9 g polyol and 9 g diisocyanate.

The MA foam was prepared by mixing the polyol with CIPs using a mechanical stirrer at a constant speed of 550 rpm for 20 s. Then, the diisocyanate was poured into the mixture, which was stirred again for another 20 s at the same speed as before. This final mixture was then poured into a smooth cylindrical polyvinyl chloride mold for the foaming process, where it was cured for 24 h at room temperature.

The morphological structure of the samples was analyzed at various pressures using a scanning electron microscope (SEM) (JEOL JSM-IT300LV, Japan). Before performing the analysis, the samples were coated with gold to obtain a clear image. The microscope was set at a voltage of 10 kV and with a high resolution across magnifications of 100×, 1000× and 3000×.

Meanwhile, the strain and rheological properties of the MA foam were tested under a shear oscillation mode using a rheometer (model MCR302, manufactured by Anton Paar) and following a similar procedure as in other studies [9,13]. A circular sample, with a diameter of 20 mm and thickness of 1 mm, was placed between the parallel plates of the measuring geometry. Magnetic field excitations in the range of 0 to 0.8 Tesla (T) were achieved by varying the current between 0 and 5 A during the tests. For the rheological properties of the MA foam, the shear strain used was under a linear viscoelastic limit (0.001 to 0.1%) with a constant frequency of 1 Hz, while for magnetostriction (elongation), the characterization was examined under zero and 0.001% of shear strain. All the changes in the length were calculated from the difference between the initial and the final gaps in the samples.

## 3. Results

### 3.1. Mechanical Properties

**Figure 1:**
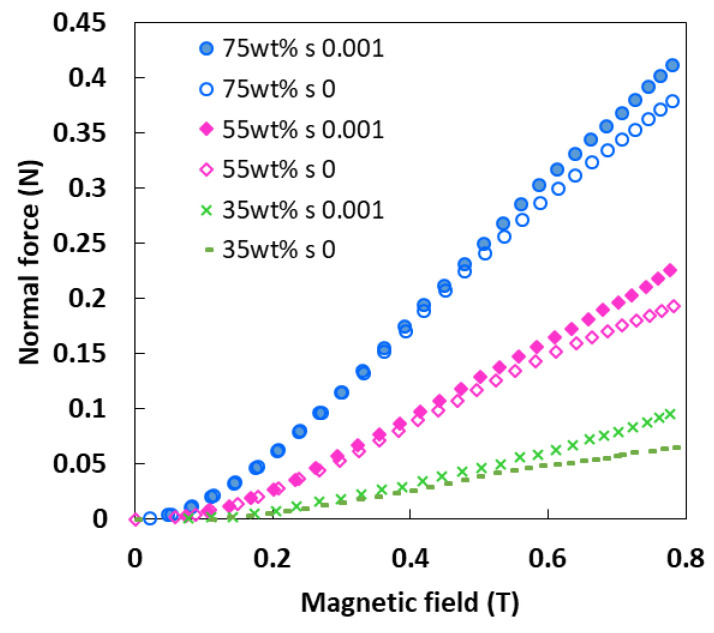
Comparison of normal forces versus the magnetic field at different strain conditions with various concentration of carbonyl iron particles (CIPs).

A soft sensor generally requires a system that can generate a reaction force to interact with the environment or to make a movement. In the MA foam under investigation, when the samples were exposed to a magnetic field, magnetization was induced in the CIPs. Simultaneously, the magnetic moments of the CIPs rearrange themselves so as to be realigned in the direction of the external magnetic field. This created interaction between the CIPs and produced a chainlike structure. At the same time, an induced/reaction force was produced, which is also known as a normal force. Thus, to understand the reaction force, the normal force was studied under two shear strain conditions, namely, zero shear strain and 0.001% shear strain, and the graph of the normal forces in various magnetic fields was plotted, as shown in Figure 1.

The graph demonstrated that all the MA foam samples exhibited a linear increase in the normal force under the influence of a magnetic field. The MA foam with 75 wt.% of CIPs produced the highest normal force, followed by the MA foams with 55 wt.% and 35 wt.% of CIPs under both shear strain conditions. All the samples produced higher normal forces with a shear strain of 0.001% than with a shear strain of zero, especially when the magnetic field intensity was larger than 0.4 T. Under a shear strain, the deformation of the samples enhanced the interaction between the CIPs and led to an increase in the normal force. The MA foam with 75 wt.% of CIPs showed a high sensitivity towards the normal force, which was as small as 0.48 mN at 0.05 T, with an improvement of 97% in sensitivity compared to 20 mN in a previous study [20] using a superflexible patterned skin film. Generally, a sensitive normal force is required, especially in soft sensors in medical applications, where a highly accurate force sensor is required during operations.

**Figure 2:**
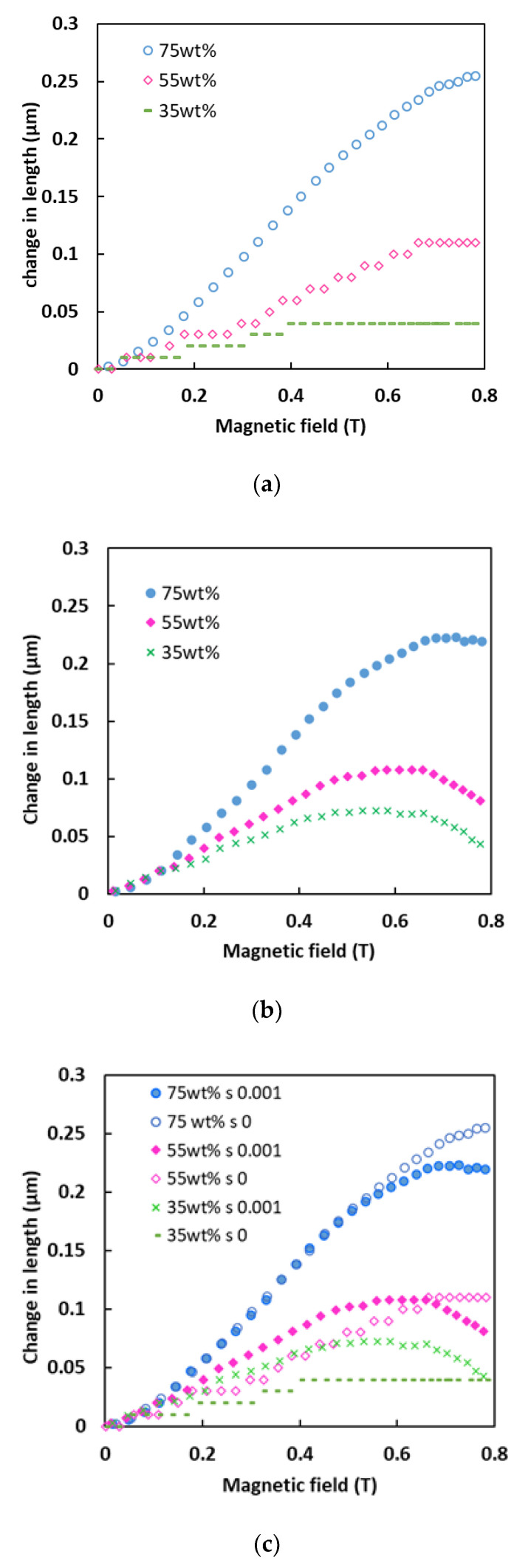
Change in length versus magnetic field at (**a**) zero, (**b**) 0.001% of shear strain, and (**c**) comparison between zero and 0.001% of shear strain.

Figure 2 shows the change in length of the MA foams measured as a function of the magnetic field intensity at (a) zero and (b) 0.001% shear strain. All the MA foams showed a growth trend in terms of the change in length in the presence of a magnetic field, indicating that the samples were elongated. In Figure 2a, the MA foams with 35 wt.% and 55 wt.% of CIPs showed a unique trend, whereby there were stepwise increases in the elongation with increases in the magnetic field intensity, before saturation lengths of 0.04 µm and 0.11 µm were reached at 0.4 T and 0.65 T, respectively. This phenomenon occurred because the normal forces that were induced were insufficient to overcome the elastic energy barrier in each mesoscopic region which possessed its own yield energy/distribution energy to elongate. On the other hand, the MA foam with 75 wt.% of CIPs showed a dramatic increase in length with an increase in the magnetic field intensity, before tending to reach saturation when approaching 0.8 T, with a change in length of 0.26 µm. The MA foam with 75 wt.% of CIPs experienced a change in length in the magnetic field at a rate of 3.76 nm/mT, which indicated a greater sensitivity of 85% compared to the rate of 25.5 nm/mT, as reported previously [14].

Meanwhile, Figure 2b demonstrates the effect of a shear strain of 0.001% on the length of the MA foams. The foams showed an enhancement in elongation with a small incremental trend in the magnetic field intensity of below 0.15 T. For the MA foam with 35 wt.% of CIPs, it was apparent that the increased change in length continued until saturation was reached at 0.07 µm with a magnetic field of 0.5 T before starting to decrease. A similar pattern was also observed for the MA foam with 55 wt.% of CIPs, except that the saturation point was at 0.11 µm with a magnetic field of 0.6 T, which was higher than that of the MA foam with 35 wt.% of CIPs. Meanwhile, for the MA foam with 75 wt.% of CIPs, the change in length started to saturate at 0.7 T, with the highest elongation being 0.23 µm compared to the MA foams with 35 wt.% and 55 wt.% of CIPs, before decreasing when the magnetic field intensity was larger than 0.7 T. In general, the decrease in length of all the MA foams might be attributed to the increase in the force of attraction between the CIPs, which reduced the repulsive force for elongation. Figure 2c shows a comparison of all the MA foams at shear strains of zero and 0.001%. The MA foams with 35 wt.% and 55 wt.% of CIPs under a shear strain of 0.001% showed larger elongation values compared to those under a shear strain of zero. This might have been due to the increased interaction between the CIPs on being subjected to the shear force, where both categories of MA foams had a low concentration of CIPs. The increase in length in these MA foams was lower because in the presence of a shear force, there was more space for the CIP particles to align. However, this phenomenon did not occur in the MA foam with a higher particle concentration of 75 wt.%. Thus, for the MA foam with 75 wt.% of CIPs, a similar increase in elongation under both shear conditions was observed at a magnetic field intensity of below 0.6 T, which could be attributed to the same effect on the interaction between the CIPs. At higher magnetic fields, the shear force that acted to produce the shear strain interrupted the interaction between the CIPs and simultaneously, reduced the elongation of the MA foam. Even though all the MA foams showed an increase in the change in length with an increasing magnetic field, it was noticed that only the MA foam with 75 wt.% of CIPs had a high sensitivity with a change in length, also known as the magnetostriction effect, with and without a shear strain.

**Figure 3:**
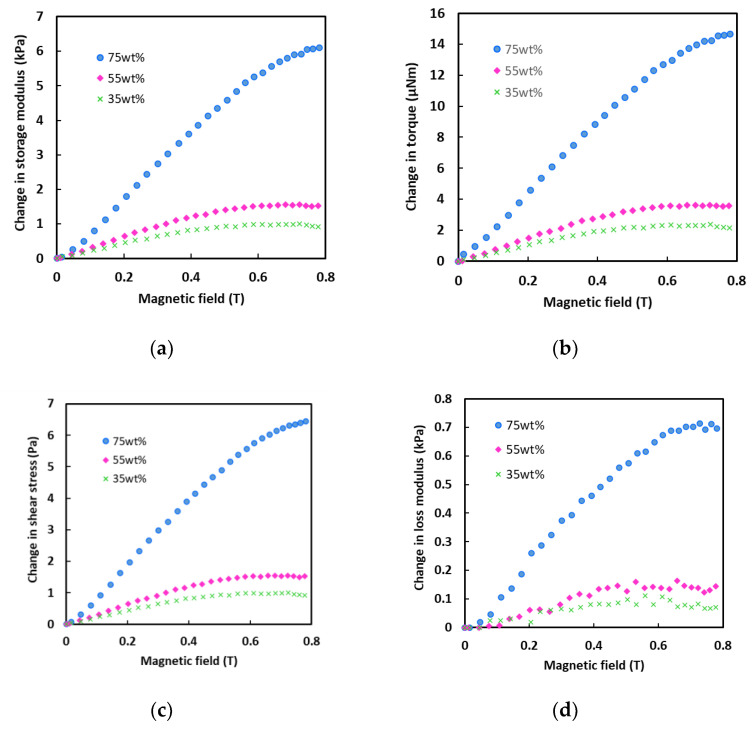
Change in (**a**) storage modulus, (**b**) torque, (**c**) shear stress and (**d**) loss modulus of magnetoactive (MA) foams versus magnetic field at 0.001% shear strain and frequency of 1 Hz.

A conventional robotic gripper commonly consists of a variety of proprioceptive sensors, such as a Hall effect sensor, to produce displacement, and a torque sensor to provide a shear force, to gather information about a subject [21]. Therefore, further analyses on the storage modulus, torque and shear stress of the MA foams were performed under a shear strain of 0.001%, as demonstrated in Figure 3a–c, respectively. The storage modulus in Figure 3a showed an increased change in length as the magnetic field increased, where the values also increased with increasing CIP concentrations. The storage modulus is an indication of the ability of an MA foam to store deformation energy in an elastic manner [22]. It is directly related to the strength of the internal chainlike structure of the CIPs in a magnetic field, where the higher the degree of the chains, the greater will be the storage modulus. Thus, this chainlike structure of the CIPs affected the torque in the MA foam during shear. Moreover, as shown in Figure 3b, the torque increased as the strength of the magnetic field increased. When the magnetic field intensity increased, the strength of the CIP chains increased and resisted the given shear force, thus producing a larger torque. The smallest and the highest torque values were obtained by the MA foams with 35 wt.% and 75 wt.% of CIPs, respectively. The strength of the CIP chains increased when the concentration of CIPs rose due to an increase in the interaction of all magnetic moments in the MA foam. Meanwhile, the results in Figure 3c showed a similar pattern, where the shear stress depended on the strength of the magnetic field. It was also observed that the shear stress had a greater effect when the concentration of CIPs increased due to strong interactions from a larger mass. Figure 3d shows that the MA foam samples had a loss modulus. Overall, the loss modulus of the MA foams increased with an increase in the magnetic field strength and CIP concentration. As the concentration of CIPs increased, the loss modulus increased along with the internal friction. The loss modulus occurred because energy was lost by the CIPs and matrix during deformation by the shear strain. However, the modulus curves were not in a smooth line as the energy was lost at different rates according to the rate of destruction and reformation of the structure, and conditioning by the internal friction.

All the MA foams showed a similar trend, where the storage modulus, torque and shear stress increased with increases in the intensity of the magnetic field. Figure 3 revealed that the MA foam with 75 wt.% of CIPs experienced significant changes to its storage modulus, torque, and shear stress compared to the other two MA foam samples of 35 and 55 wt.% of CIPs. In other words, all the changes to the storage modulus, torque and shear stress properties demonstrated that the MA foam with 75 wt.% of CIPs had a stronger magnetoelastic effect compared to the MA foam with 35 and 55 wt.% of CIPs. The sensitivities of the storage modulus, torque, and shear stress of the MA foam with 75 wt.% of CIPs were 8.97 Pa/mT, 0.021 µN/ mT and 0.0096 Pa/mT, respectively. From the perspective of the gripping capability, the MA foam with 75 wt.% of CIPs exhibited a strong performance and multiple sensitivities that enhanced the capability of the foam.

**Figure 4:**
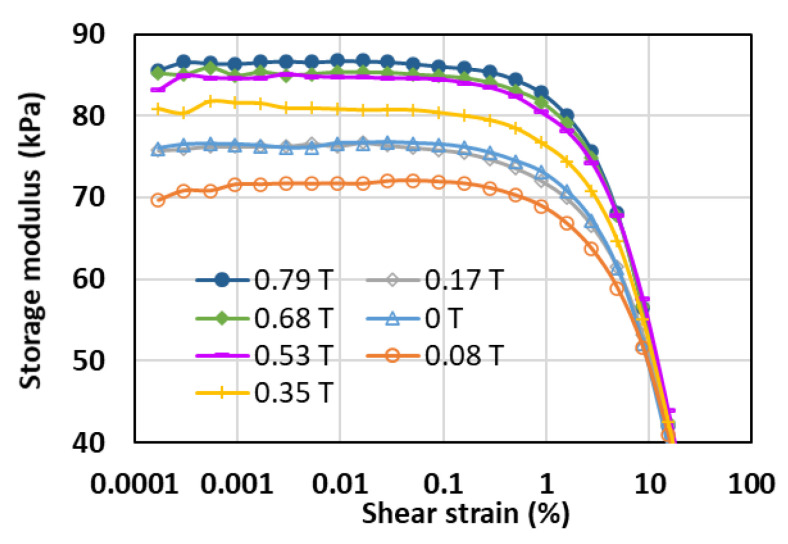
Storage modulus versus shear strain at different magnetic field strengths and frequency of 1 Hz.

Therefore, the MA foam sample with 75 wt.% of CIPs was selected for further analysis to study the effects of various parameters. Figure 4 illustrates the storage modulus of the MA foam with 75 wt.% of CIPs at different magnetic field intensities. At the given magnetic field intensity of 0.08 T, a decrease in the storage modulus was detected due to the small magnetic field stimulus, where the CIPs started to vibrate and, thus, lose the polymer matrix, making the foam softer. Then, the storage modulus increased together with an increase in the magnetic field intensity. This time, the gain in the magnetic field intensity strengthened the reaction between the neighboring CIPs, thereby producing an interaction that resulted in the formation of internal chainlike structures. As the magnetic field increased, the chainlike structures became stronger and increased the elasticity of the MA foam, while simultaneously increasing the storage modulus. A plateau region was observed for all the MA foam samples at a low shear strain of 0.0001 to 0.06%. This plateau region started to slowly decrease until the shear strain was approximately 1%, and then, rapidly decreased further at a higher shear strain (larger than 1%). The plateau region represented the linear viscoelastic (LVE) region, where the range of shear strain force could be applied to the sample for any mechanical or rheological tests [23]. Generally, in this region, the deformation of the sample is within the range of the elastic limit and the sample will be able to recover to its initial form. However, if a larger shear strain is applied beyond the LVE region, the deformation of the sample will be permanent, and the sample might not be able to return to its original form once the polymer matrix has lost its elasticity. Figure 4 shows that there was a slight decrease in the LVE region of the MA foam with 75 wt.% of CIPs with an increase in the magnetic field intensity due to the strong bonds between the CIPs in the presence of the magnetic field. It was interesting to note that the LVE region for the MA foam with 75 wt.% of CIPs with various magnetic field intensities was in the range of 0.001 to 0.1%. Thus, the LVE region of 0.001 to 0.1% was chosen for further analysis.

**Figure 5:**
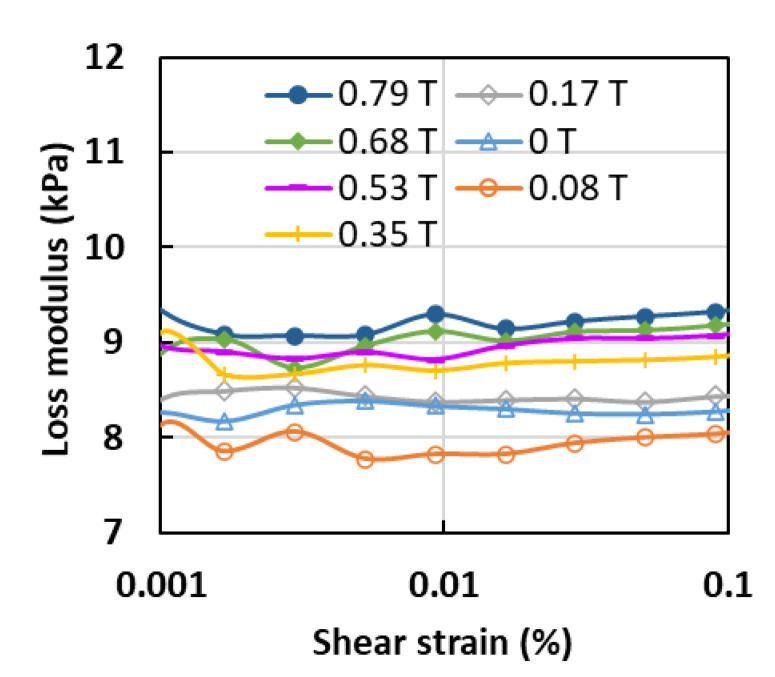
Loss modulus versus shear strain at different magnetic field strengths and constant frequency of 1 Hz.

Figure 5 depicts the relationship between the loss modulus and the change in shear strain at different magnetic field intensities and under a constant frequency of 1 Hz. The loss modulus within the LVE region was almost constant with changes to the shear strain, which indicated that the shear strain did not affect the amount of dissipated energy. The graph showed the lowest value of loss modulus was obtained when the magnetic field strength was increased to 0.08 T, which was lower than the loss modulus value at 0 T. This value could be explained by the same phenomenon as previously, where the decrease in loss modulus was closely connected with the low storage modulus of the MA foam with 75 wt.% of CIPs. At the same time, the elastic property was low, and thus, the low resistance that was obtained during the shear strain affected the loss modulus. However, later, with a further increase in the magnetic field strength from 0.17 to 0.79 T, the loss modulus also increased with a similar trend.

**Figure 6:**
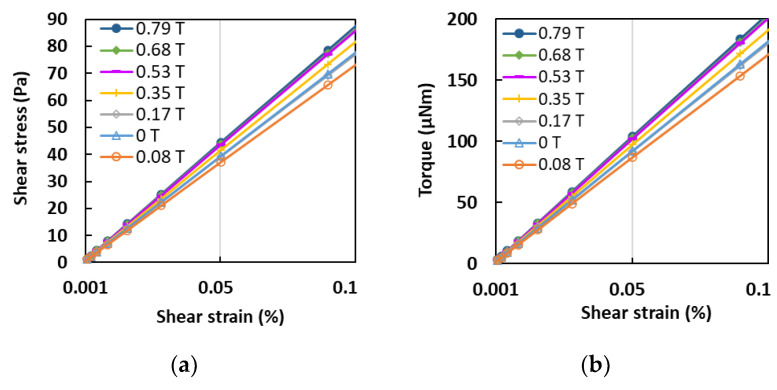

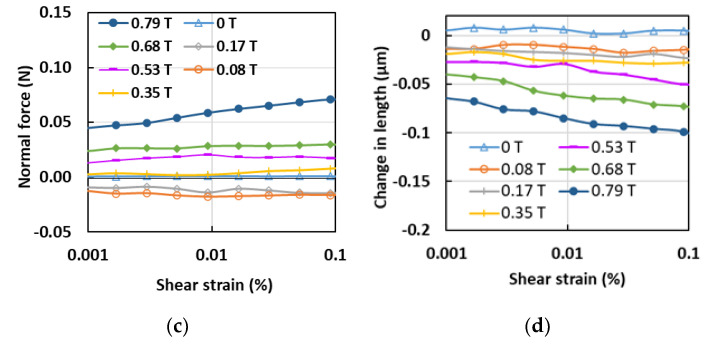
Relationship of (**a**) shear stress, (**b**) torque, (**c**) normal force and (**d**) change in length with shear strain at different magnetic field strengths.

Further analyses into the effects of the shear strain on the MA foam with 75 wt.% of CIPs are shown in Figure 6. Figure 6a shows the effect of the shear stress on the MA foam with 75 wt.% of CIPs when the shear strain was increased. It shows that the shear stress had a linear relationship with the shear strain, thereby indicating its elastic region. The gradient of the shear stress graph represented the modulus rigidity, which is the elasticity or flexibility of the MA foam. When a magnetic field intensity of 0.08 T was applied, the value of the shear stress decreased compared to the value of the shear stress at 0 T. There was a low interaction between the CIPs inside the sample induced by a small force, and these began to vibrate. Thus, it was easier for the applied shear strain to deform the sample compared to the magnetic field intensity at 0 T, where the particles were locked in the matrix. Then, as the magnetic field intensity increased, the shear stress started to increase with an increase in the shear strain. The shear stress of the MA foam was higher when the magnetic field intensity was increased due to the strong interaction between the particles at high CIP concentrations. A similar trend was also observed in the torque values, as shown in Figure 6b. Torque is the measurement of the force that can cause an object to acquire an angular acceleration. As demonstrated in Figure 6b, the torque increased with an increase in the shear strain as the magnetic field intensity was increased from 0.17 T to 0.79 T. However, a similar trend was observed at a magnetic field intensity of 0.8 T, where the torque was smaller compared to the value at 0 T due to the low elasticity that affected the torsion, as discussed previously. The MA foam was able to control the shear stress and torque since both properties were linearly dependent on the shear strain. This linear region is needed as an ideal region for potential applications since the behavior can be easily predicted with changes to the shear strain.

Figure 6c shows the variation in the normal force with the applied shear strain at different magnetic field intensities. Interestingly, the values of the normal force were observed to drop as the magnetic field intensity was increased up to 0.17 T, while, in contrast, the normal force increased with further increases in the magnetic field intensity up to 0.68 T. In the beginning, a decline in the normal force was due to the smaller magnetic force that was induced compared to the elastic and shear forces. With further increases in the magnetic field intensity, the magnetic force induced by the CIPs was increased to withstand the elastic and shear forces. However, the values of the normal force were almost constant with changes to the shear strain for magnetic field intensities beginning from 0 to 0.68 T, thereby showing that the shear strain was independent. Nevertheless, there was a dramatic increase in the normal force at the highest magnetic field intensity of 0.79 T. This unique pattern might have been due to the strong relationship between the external magnetic field and the CIPs, which caused them to align their magnetic moments in the direction of the external magnetic field. Thus, this simultaneously resulted in an increase in the induced normal force with an increase in the shear strain. Meanwhile, Figure 6d depicts the relationship between the change in length versus the shear strain. No significant change in length was observed in the MA foam sample with shear strain at a magnetic field intensity of 0 T as there was very little rebound from the foam matrix. All the samples showed negative values, which represented a contraction due to the magnetostriction phenomenon. However, as the magnetic field intensity was increased from 0 to 0.35 T, the change in length was larger but remained constant along with the shear strain. The deformation to the MA foam was very much influenced by the presence of a magnetic field rather than the shear strain. However, when the magnetic field was increased to 0.53 T, the change in length of the MA foam increased along with an increase in the shear strain, which could be correlated to the increased contraction that occurred in the MA foam. A similar trend was observed at magnetic field intensities of 0.68 and 0.79 T, which showed increases in the contraction values. Consequently, the contraction in the MA foam with 75 wt.% of CIPs exhibited a negative magnetostriction effect under continuous increases in the shear strain. This might have been due to the arrangement of the CIPs in the surrounding magnetic field under a shear strain.

**Figure 7:**
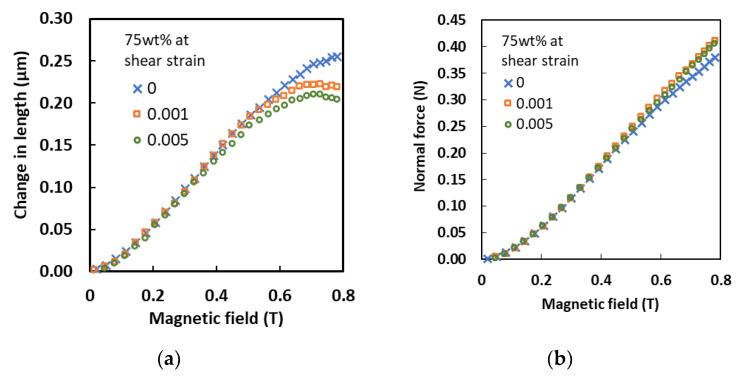
(**a**) Change in length and (**b**) normal force under various magnetic fields at different shear strains.

The effect of variable shear strain values on the MA foam with 75 wt.% of CIPs at different magnetic field intensities was examined. Figure 7a depicts the change in length produced in the MA foam at different shear strains. It was observed that a similar trend occurred with magnetic field intensities of less than 0.4 T for all shear strain conditions.

At zero shear strain, the elongation increased linearly with an increase in the magnetic field intensity until it became almost constant after 0.7 T. Meanwhile, under a shear strain of 0.001%, the elongation increased with an increase in the magnetic field intensity up to 0.5 T, and slowly decreased at magnetic field intensities of more 0.7 T. A similar effect was also observed with a shear strain of 0.005%, where the elongation of the MA foam steadily increased until a magnetic field intensity of 0.5 T was achieved, reached a maximum at 0.7 T, and then, a slow reduction in the length was observed. Concurrently, a decrease in the shear strain resulted in a reduction in the elongation of the MA foam due an increase in the forces of attraction between the CIPs. However, the opposite was observed with regard to the behavior of the normal force, as plotted in Figure 7b. The increase in shear strain (0.001 and 0.005%) led to an increase in the normal force of the MA foam compared to the values at zero shear strain, obviously after being stimulated by a magnetic field strength of more than 0.5 T.

**Figure 8:**
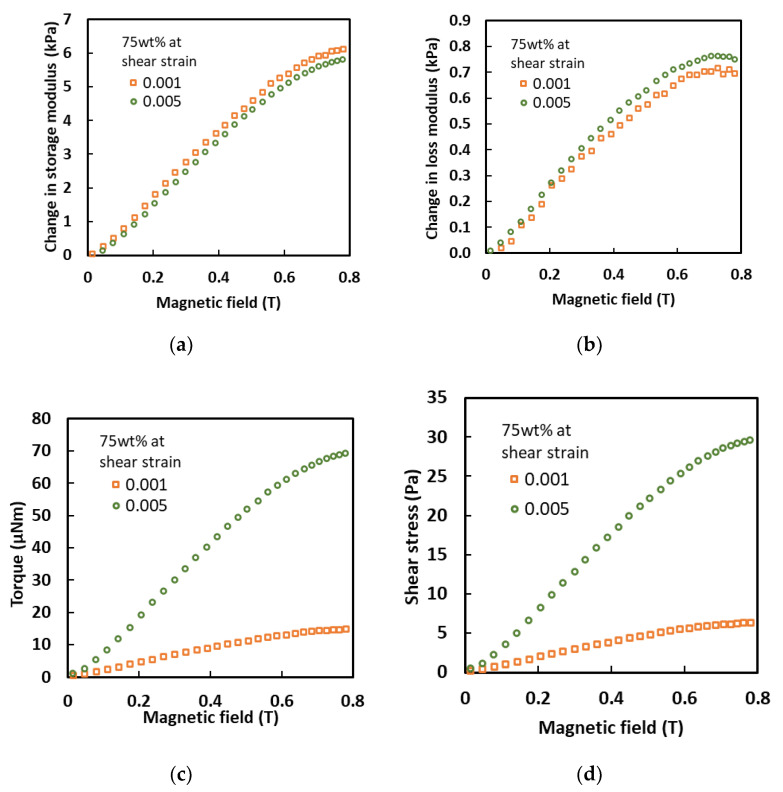
Relationship of (**a**) storage modulus, (**b**) loss modulus, (**c**) torque and (**d**) shear stress with various magnetic fields at different shear strains.

Figure 8a,b demonstrate that there was a linear increase in the of storage and loss moduli with the magnetic field until they started to reach saturation at 0.6 T. The change in the storage modulus for a shear strain of 0.001% by increasing the magnetic field was higher compared to a shear strain of 0.005%. In contrast, the change in the loss modulus was in the reverse pattern, where the change in the loss modulus was higher at a higher shear strain. The difference in values might have been due to the high resistance produced by the aligned solid CIP particles embedded in the MA matrix, which acted as a barrier to the shear strain. Thus, the presence of the CIPs reduced the storage modulus and increased the loss modulus. Moreover, the effects of the torque and shear stress were also observed, and were graphically presented in Figure 8c,d, respectively. Overall, a shear strain of 0.001% resulted in larger torque and shear stress effects compared to a shear strain of 0.005%. A larger torque occurred, as shown in Figure 8c, due to an increase in the shear strain caused by the large displacement of the matrix, which was needed since the CIPs were aligned in the direction of the magnetic field. Furthermore, a larger shear stress at a higher shear strain was also obtained to demonstrate that the MA foam with 75 wt.% of CIPs could be amplified within the LVE region. It could be seen that the MA foam with 75 wt.% of CIPs had a linear dependency with regard to the magnetostriction, normal force, storage modulus, loss modulus, torque and shear stress, making it possible for these to be controlled by changing the intensity of the magnetic field for sensor applications.

### 3.2. Morphology Property

**Figure 9:**
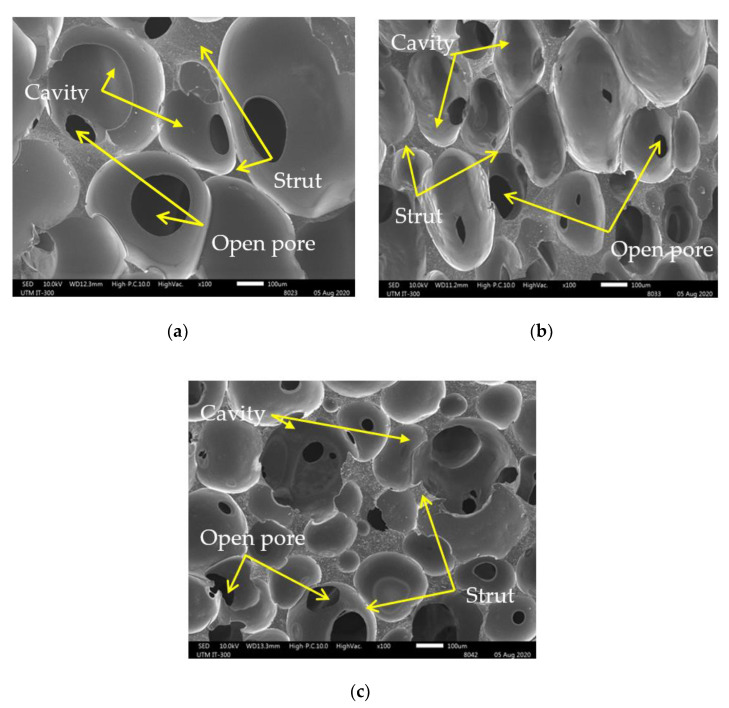
SEM images with magnification 100× of (**a**) polyurethane (PU) foam, (**b**) 55 wt.% and (**c**) 75 wt.% of CIPs within MA foams.

**Figure 10:**
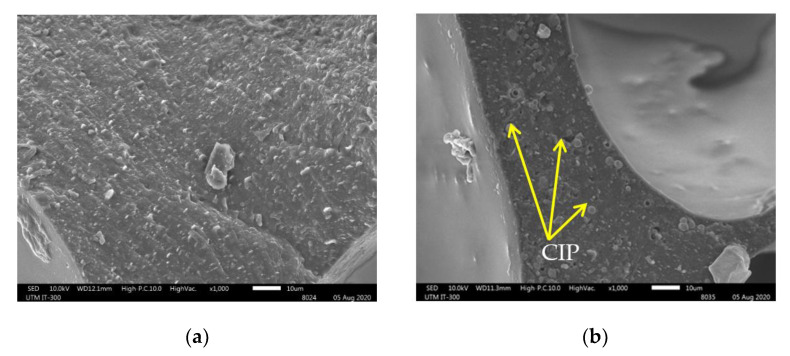

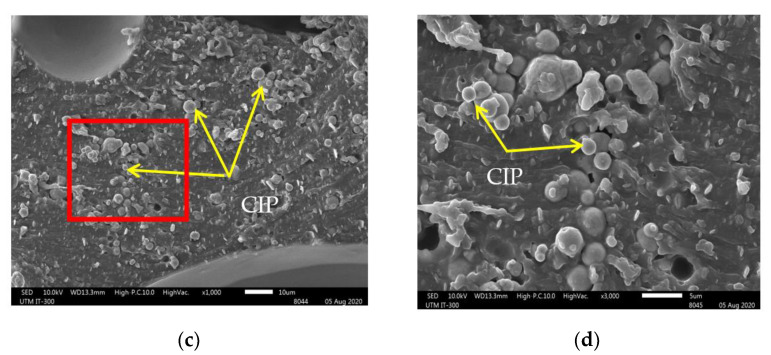
SEM images with magnification 1000× of strut of (**a**) PU foam, (**b**) 55 wt.%, (**c**) 75 wt.% of MA foams and (**d**) extrapolated with magnification 3000× from the red box in (**c**).

Figure 9 shows a comparison of the morphologies of (a) PU foam, (b) MA foam with 55 wt.% of CIPs, and (c) MA foam with 75 wt.% of CIPs. The PU foam had large and nonuniform cavities, with an average size of 585.7 ± 97.5 µm. The average size of the cavities was reduced as the CIP concentration was increased, where the average size of the cavities in the MA foam with 55 wt.% of CIPs was 440.1 ± 105.8 µm, while the average size of the cavities in the MA foam with 75 wt.% of CIPs was 403.7 ± 122.8 µm. However, the PU foam and MA foam with 55 wt.% of CIPs had more closed pores compared to the MA foam with 75 wt.% of CIPs, which showed an increase in the number of open pores. The cavities in the MA foam with 75 wt.% of CIPs were deeper and had a three-dimensional connectivity like a tunnel compared to the MA foam with 55 wt.% of CIPs. The deeper cavities in the MA foam with 75 wt.% of CIPs was due to the increased concentration of CIPs, which simultaneously led to an increase in the number of cavities (bubble nucleation). Since expansion was difficult with the presence of more CIPs, the bubbles that coalesced with other bubbles were deeper and formed connecting cavities. Thus, this finding indicated that when the concentration of CIPs increased, the depth of the cavities also increased. Meanwhile, the reduced size of the cavities might have been because more matrix was used to embed the CIPs, and this restricted the expansion of the cavities. In other words, the presence of more CIPs hindered the expansion of the cavities. Figure 10a shows the SEM image of a PU foam strut without CIPs, while Figure 10b,c show the SEM images of struts of MA foams with 55 and 75 wt.% of CIPs. The presence of spherical particles, which was attributed to the CIPs embedded in the PU matrix (strut), was observed in the MA foams with 55 wt.% and 75 wt.% of CIPs compared to the PU foam. The higher concentration of CIPs in the MA foam with 75 wt.% of CIPs reduced the distance between the CIPs, thereby leading to the formation of compact struts compared to the MA foam with 55 wt.% of CIPs, as shown in Figure 10d.

## 4. Discussion

**Figure 11:**
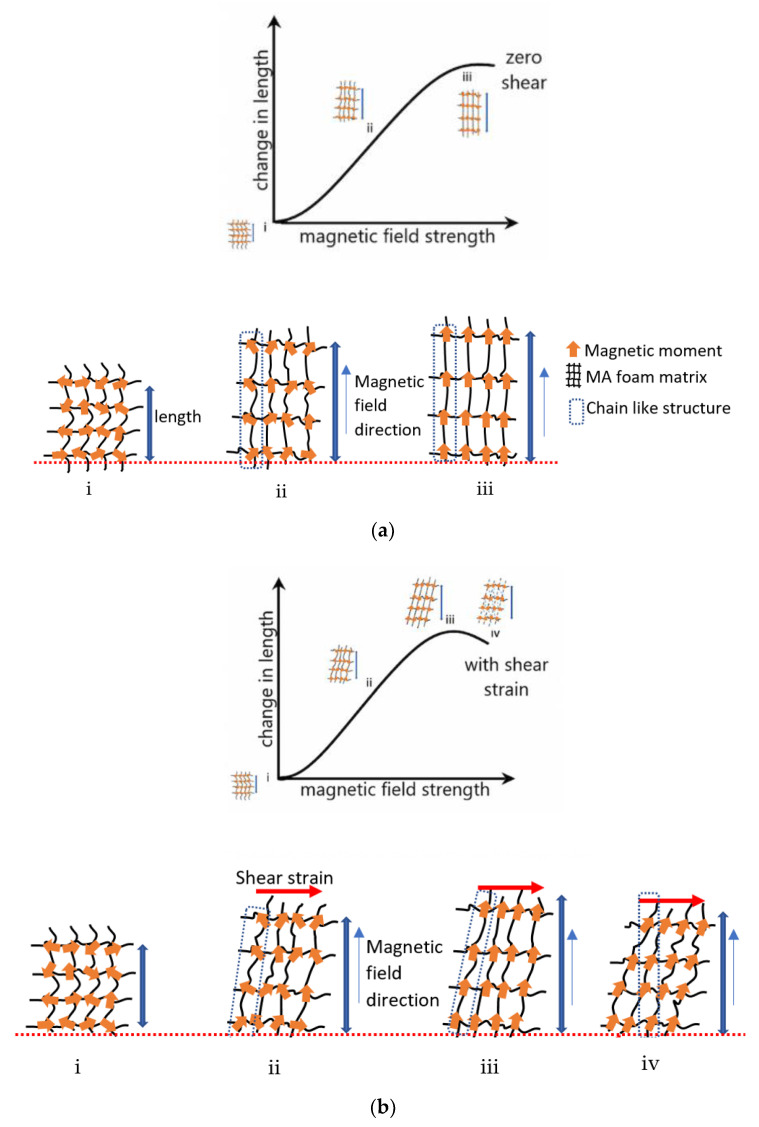
Change in length of MA foam under (**a**) zero and (**b**) 0.001% shear strain with schematic physical property diagram transition.

The MA foam matrix consisted of a soft segment and hard segment, which were flexible within their elastic limits. The matrix and CIPs were able to accommodate the presence of a magnetic field and shear strain to enable the foam to reach its new state of equilibrium. Figure 11a shows the graph of the change in length against the magnetic field at zero shear strain, with the schematic diagram of the transition of the physical properties from regions (i) to (iii), with the following mechanisms accordingly:

(i) At zero magnetic field, the matrix and CIPs of the MA foam were in a state of equilibrium, while the magnetic moments were in a random direction.
(ii) At the beginning of exposure to a magnetic field, the external magnetic field induced a magnetic moment in each CIP. The magnetic moments rotated to enable the CIPs to align themselves with the external magnetic field and to induce a force within each CIP. Therefore, the interaction between the CIPs caused them to develop an internal chainlike structure within the MA foam to generate repulsive and attractive forces. The total forces caused the soft segment in the MA foam matrix to stretch, bend, and twist. Since the attractive force induced was larger than the repulsive force, the MA foam was elongated and magnetostriction occurred.
(iii) At a high magnetic field intensity, the increase in the induced forces produced a larger repulsive force, and a larger elongation occurred. When the CIPs reach saturation point, where all the magnetic moments were aligned with the external magnetic field, a constant change in length with no further elongation in the MA foam occurred.

Figure 11b shows the change in length with various magnetic fields at a constant shear strain, where the schematic diagrams (i) to (iv) show the transition of the physical properties of the MA foam in relation to the graph. The details of each transition were as follows:

(i) At zero magnetic field, the matrix and CIPs of the MA foam were in a state of equilibrium with the magnetic moments in a random direction, as shown in Figure 11b(i)
(ii) At the beginning of exposure to a magnetic field with a constant shear: Figure 11b(ii) shows that the interaction between the induced magnetic moments produced a chainlike structure in the MA foam. Due to the shear force, the interaction between the CIPs increased, thus, causing the repulsive force to increase and to provide a larger elongation compared to a zero shear.
(iii) With an increase in the magnetic field, as shown in Figure 11b(iii), the internal chainlike structure made up of CIPs in the MA foam was stronger. This strength resulted in the maximum elongation of the MA foam with further increases in the magnetic field strength due to the shear strain. The chainlike structure tilted, and the strut could have been bent, stretched or contracted, thereby reducing the elastic energy boundary.
(iv) At a high magnetic field, the chainlike structure was strongest and thus, a larger shear force was needed to produce a shear strain of 0.001% to consequently break this structure. The matrix and CIPs were displaced, and the CIPs established a new chainlike structure together with other nearby CIPs in the MA foam, as can be seen in Figure 11b(iv). Thus, the structure produced a larger attractive force compared to the repulsive force to gain a new state of equilibrium. Finally, the attractive force caused a contraction in the MA foam, and a simultaneous decrease in elongation was observed.

**Figure 12:**
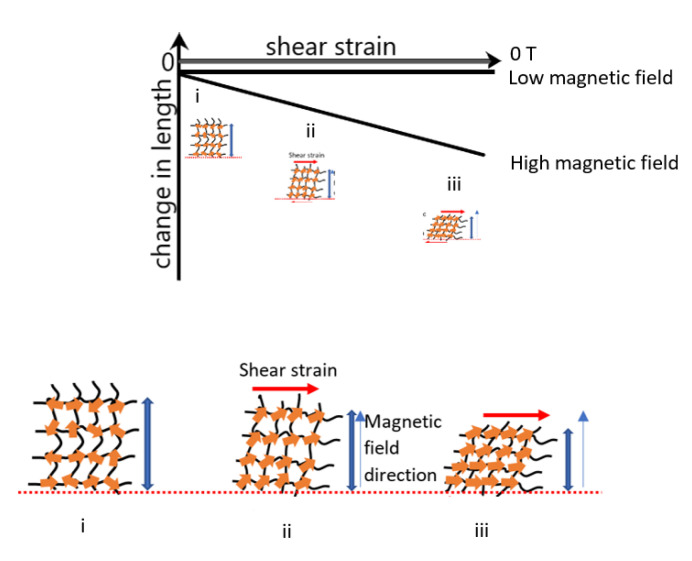
Change in length of MA foam under various shear strains at different magnetic field condition of zero, low and high magnetic fields.

Figure 12 demonstrates the arrangement of the CIPs to further explain the effect of the shear strain at various magnetic field intensities. Initially, it was observed that the CIPs and matrix slipped between each other but with no change in length. However, at a low magnetic field, a magnetic dipolar interaction was produced, and this caused a contraction to occur. The increase in the shear/displacement of the matrix did not affect the change in length to induce a force and thus, a constant contraction length was obtained. Meanwhile, at a high magnetic field intensity, the transition in the physical properties of the MA foam, as shown in Figure 12 (i) to (iii), was explained with the following details:

(i) At zero magnetic field and zero shear, the MA foam matrix and CIPs were in a state of equilibrium with the magnetic moments being in a random direction.
(ii) When a high shear strain was applied at a constant magnetic field intensity, the presence of a high magnetic field induced magnetic moments to align the CIPs in the direction of the external magnetic field. However, the magnetic moments of the CIPs started to rotate, and due to the shear effect, the matrix was displaced, and the CIPs produced a bigger attractive force than a repulsive force. Thus, a negative magnetostriction effect was observed, whereby the sample experienced a contraction.
(iii) With further increases in the shear strain at a constant magnetic field strength, as there was no change to the intensity of the magnetic field, there was no enhancement of the induction force. However, since the shear strain was increased, the CIPs were displaced, and the rearrangement of the magnetic moments produced a larger attractive force that resulted in a linear decrease in length with increases to the shear strain.

The induced force occurred in two ways; the magnetic field intensity and shear strain were swept under a constant high magnetic field. Firstly, as the intensity of the swept magnetic field increased, the interaction between the CIPs became stronger; the arrangement of the CIPs along the direction of the magnetic field improved, thereby producing a larger induced force parallel with an increase in length. Secondly, as the swept shear strain increased under a constant magnetic field, the displacement of the CIPs and matrix occurred with a larger magnetic field intensity, and, hence, a larger induced force was produced. Furthermore, when the magnetic field was applied, the magnetic moments of the CIPs were polarized, and these created the internal chainlike structure. As a shear force was applied to this structure, a torque was induced around this chain. As the magnetic field increased, the deformation of the MA foam enhanced the strength of the particle chain and produced a large torque. The CIP chain can play an important role in the measurement of the shear force in the future. In terms of the shear modulus, which is known as the storage modulus, the magnetic-induced storage modulus increased with an increase in the concentration of CIPs, and this was in good agreement with the Einstein–Guth–Gold equation

(1) $$G^{\prime }=G_{0}(1+2.5Ø\;+14.1{Ø}^{2})$$

where G′ is the storage modulus, G_0_ is the storage modulus of the unfilled matrix, and ∅ is the volume fraction of the particles. It should be noted that the dramatic increase in the storage modulus of the MA foam with 75 wt.% of CIPs might be related to the structure of the foam itself, which was also related to the shear modulus. At a constant concentration, the storage modulus was affected by the magnetic dipolar interaction between the CIPs in the matrix. A stronger interaction of magnetic CIPs occurred with an increase in the magnetic field, thus leading to an enhancement of the shear modulus. The loss modulus was observed to have increased with an increase in the magnetic field intensity as well due to the energy loss that resulted from the deformation. However, it was observed that the constant dependence of the loss modulus on the shear strain revealed that there was no loss of energy at all the given strains, where the MA foam showed its capability as an energy saver under various shear conditions.

## 5. Conclusions

In this work, the multisensory and high sensitivity of MA foam with different concentrations of CIPS were examined. The MA foam had the capability to detect magnetostriction, shear and loss modulus, torque and normal force. The optimum magnetostriction effect was observed at 75 wt.% of CIPs. Based on the concentration and structure of the MA foam, the capability to detect small changes in length and normal force was enhanced. The normal force sensitivity was 0.48 mN at 0.05 T, with an improvement of almost 97% in sensitivity. Meanwhile, the magnetostriction effect on sensitivity was enhanced to nanoscale at 3.76 nm/mT with an enhancement of 85%. The sensitivities of the storage modulus, torque and shear stress of the MA foam with 75 wt.% of CIPs were 8.97 Pa/mT, 0.021 µN/ mT and 0.0096 Pa/mT, respectively.

The MA foam behavior can be described as below:

(i) The normal force was strongly dependent on the concentration of CIPs and the intensity of the magnetic field for both conditions of shear strain due to a strong interaction between the CIPs.
(ii) The magnetostriction behavior linearly increased with the magnetic field at a high concentration of CIPs because of the strong repulsive force produced by the CIPs in the matrix. At a low concentration of CIPs, the amount of force produced was not sufficient to overcome the elastic force. It was also observed that under a constant shear strain applied at a high magnetic field strength, the attractive force increased since the displacement of MA foam was reduced.
(iii) Under the application of a constant shear strain, the storage modulus, loss modulus, torque and shear stress increased linearly with increases in the intensity of the magnetic field.
(iv) Under different magnetic field intensities, the normal force remained constant as a swept shear strain was applied. However, at a high magnetic field strength, the normal force increased with an increase in the shear strain. It was believed that during the swept shear strain, the matrix and CIPs were displaced in the MA foam. Thus, some changes in the magnetic polarization of the matrix led to an increase in the attractive force. This phenomenon resulted in the occurrence of a negative magnetostriction effect during the swept shear strain.

This study contributes both to the improvement of the sensitivity of MA foams and an understanding of the fundamental effect of the shear strain on MA foams. Significant improvements to the proposed MA foam will give it the potential to be used in soft sensor applications.

## Figures and Tables

**Table 1:** Selected studies investigating the different mechanical properties of the magnetoactive (MA) foams under magnetic fields.

Test	Manipulated Variable	Authors
Compression	Strain 5%	Davino et al. [8] (p. 1)
Compression	Strain 5%	D’Auria et al. [10] (p. 1)
Compression	Strain 1, 2, 3 & 4%	Volpe et al. [11] (p. 1)
Storage modulus	Shear strain 0.3%	Gong et al. [9] (p. 1)
Storage modulus	Shear strain 0.01%	Ju et al. [16]
Storage modulus	Shear strain 0.01%	Plachy et al. [12] (p. 1)
Storage modulus	Shear strain 0.001%	Muhazeli et al. [13] (p. 1)
Magnetostriction	Magnetic field 200–1000 mT	Wang et al. [14] (p. 1)
Magnetostriction	Magnetic field 1000–8000 mT	Bednarek et al. [15] (p. 1)

## Data Availability

Data available on request.
